# Emergence of Dengue Virus Serotype 3, Lineage III_B.3.2, Angola

**DOI:** 10.3201/eid3111.251079

**Published:** 2025-11

**Authors:** Jocelyne Neto de Vasconcelos, Ingra M. Claro, Raissa Heloisa de Araujo Eliodoro, Filipe R.R. Moreira, Amilton Pereira, Luzia Samuel, Esménia Coelho Rocha, Eusébio Manuel, Nelson Mapenzi-Kashali, Fiston Cikaya Kankolongo, Darlan S. Cândido, Jaqueline Goes de Jesus, Gilda Mariano, Sofia Sousa, Carina Clemente, Cláudia Muenga, Ilaria Dorigatti, William M. de Souza, Charles Whittaker, Victoria M. Cox, Wes Hinsley, Nicholas Loman, Joshua Quick, Placide Mbala, Nuno R. Faria, Joana Morais, on behalf of the FEEVIR Consortium

**Affiliations:** Centro de Investigação em Saúde de Angola, Bengo, Angola (J.N. de Vasconcelos); Ministry of Health, Luanda, Angola (J.N. de Vasconcelos, A. Pereira, L. Samuel, E. Manuel, J. Morais); Imperial College London, London, UK (J.N. de Vasconcelos, D.S. Cândido, I. Dorigatti, C. Whittaker, V.M. Cox, W. Hinsley, N.R. Faria); Universidade de São Paulo, São Paulo, Brazil (I.M. Claro, R.H. de Araujo Eliodoro, E. Coelho Rocha, J.G. de Jesus, N.R. Faria); University of Kentucky, Lexington, Kentucky, USA (I.M. Claro, W.M. de Souza); Federal University of Rio de Janeiro, Rio de Janeiro, Brazil (F.R.R. Moreira); Universidade Agostinho Neto, Luanda (E. Manuel, J. Morais); Institut National de Recherche Biomédicale, Kinshasa, Democratic Republic of the Congo (N. Mapenzi-Kashali, F. Cikaya Kankolongo, P. Mbala); Bahiana School of Public Health, Bahia, Brazil (J.G. de Jesus); Cligest Clinic, Luanda (G. Mariano, S. Sousa, C. Clemente, C. Muenga); University of Birmingham, Birmingham, UK (N. Loman, J. Quick); Université de Kinshasa, Kinshasa (P. Mbala)

**Keywords:** dengue virus, serotype 3, arbovirus, viruses, Angola, epidemiology, flavivirus, suitability, mobility, surveillance, mosquito-borne infections, vector-borne infections

## Abstract

We detected dengue virus serotype 3 in 11.8% (16/136) of febrile patients in Luanda Province, Angola, during April and July 2024. Our genetic analyses reveal that dengue virus serotype 3 lineage III_B.3.2 probably was imported from the Americas into Angola in late 2022 and then spread through local transmission.

Dengue virus (DENV) is transmitted primarily by *Aedes aegypti* mosquitoes and is the most widespread arbovirus globally (*1*). DENV is classified into 4 serotypes, DENV-1–4, each comprising several genotypes and lineages (*2*). Secondary infection with a heterologous serotype can increase disease severity through antibody-dependent enhancement (*3*).

In Africa, DENV incidence has risen sharply (*4*). Although malaria remains the dominant febrile illness, climate change might be increasing suitability for *Aedes* mosquito–borne arboviruses in the continent. In Angola, dengue became a notifiable disease in 2017. Molecular surveillance has previously confirmed the circulation of DENV-1 (2013) (*5*), and DENV-2 (2018) (*6*). In April 2024, four suspected dengue cases in Luanda Province reported to Angola’s Ministry of Health prompted an outbreak investigation.

We tested a convenience sample of 136 febrile patients (median age 33.5 years, interquartile range [IQR] 13–39 years) who visited 3 clinics in Luanda Province during April–November 2024. We tested residual diagnostic samples for DENV, chikungunya virus (CHIKV), and Zika virus (ZIKV) by using real-time reverse transcription PCR (Taqman Arbovirus Triplex Kit; Thermo Fisher Scientific, https://www.thermofisher.com/us/en/home.html.html) at the National Institute for Health Research under Angola’s National Arbovirus Surveillance program and in accordance with the National Ethics Committee of the Ministry of Health.

Of 136 samples, 16 (11.8%) were positive for DENV (Figure; Appendix Figure 1). Median cycle threshold was 29.7 (IQR 26.9–32.1), and median patient age was 31.5 years (IQR 10.5–40.5 years). None tested positive for CHIKV or ZIKV. Positive cases were geographically distributed across 3 municipalities in Luanda Province: Luanda (9/16 [56.3%]), Viana (6/16 [37%]), and Talatona (1/16 [6.3%]) (Appendix Table 1). We detected positive cases during April–July 2024 and detected no cases during August–November (dry season). Two positive case-patients required platelet transfusion, consistent with severe dengue and possible secondary infection (Appendix Table). Climatic suitability for *Ae. aegypti* mosquito transmission (index P, lagged +2 months) remained above 1 during September–July, confirming permissive conditions for transmission during the detection window (Figure; Appendix Figure 1).

**Figure:**
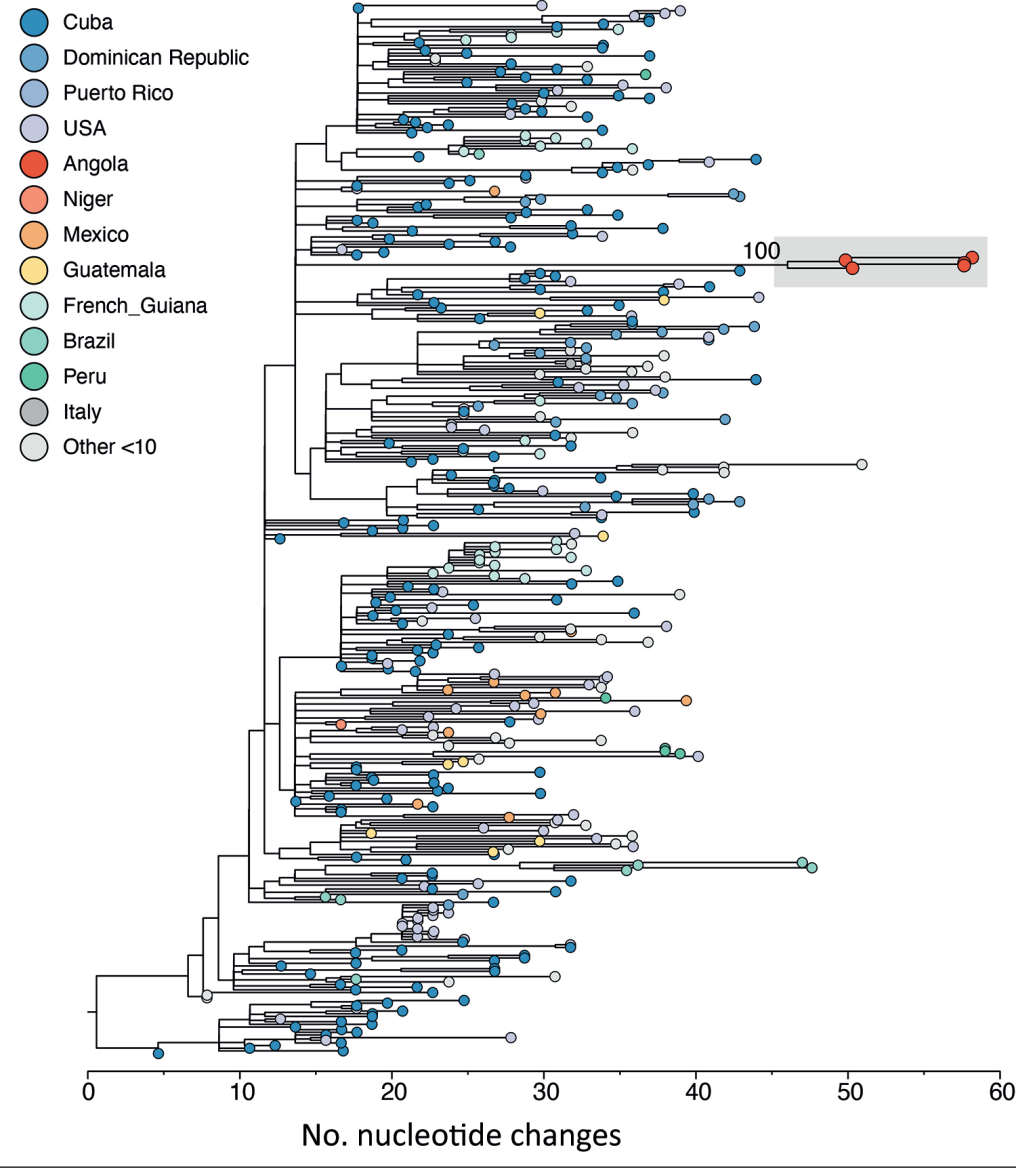
Maximum-likelihood phylogenetic tree for dengue virus serotype 3, lineage III_B.3.2, Angola. Tips are colored by country of infection. Luanda sequences are shown in red (Appendix Figures 5, 6, https://wwwnc.cdc.gov/EID/article/31/11/25-1079-App1.pdf). Countries with <10 sequences are grouped as other and include Costa Rica (n = 7), Trinidad and Tobago (n = 7), Haiti (n = 4), Guyana (n = 3), Italy (n = 2), El Salvador (n = 2), Saint Lucia (n = 1), Saint Martin (n = 1), Panama (n = 1), Niger (n = 1), and Venezuela (n = 1).

We attempted sequencing of all 16 positive samples by using a multiplex PCR protocol on the Oxford Nanopore MinION platform (https://nanoporetech.com) (Appendix). We recovered 6 near-complete and partial DENV-3 sequences (median coverage 37.6%, IQR 21.5%–57.1%) (Figure; Appendix Table). All were classified as DENV-3 lineage III_B.3.2. Lower cycle threshold values correlated with higher horizontal sequencing coverage (r ρ = –0.44; p = 0.1) (Appendix Figure 2). Maximum-likelihood phylogenetic analysis showed Angola sequences clustering into a single clade (bootstrap = 100) (Figure; Appendix). Molecular clock analysis estimated their common ancestor to be around late October 2022 (95% Bayesian CI April 2022–March 2023) (Appendix Figures 4–6).

Phylogenetic analyses revealed that Angola sequences were more closely related to viruses from the Americas (Figure; Appendix Figures 4, 5). However, undersampling and inequities in sequencing capacity could result in alternative epidemiologic scenarios, so we compared air passenger traffic into Angola (Appendix Figure 6) with lineage III_B.3.2 sampling intensity measured as the number of publicly available genomes for this lineage per million inhabitants in any given country where this lineage had been detected (according to GenBank data as of April 25, 2025). We observed a moderate correlation (Pearson r = 0.55; p = 0.042), suggesting that countries with frequent travel links, particularly Cuba, harbored closely related strains. However, those findings should be interpreted cautiously given limited recent genomic DENV-3 data from several regions, including Brazil (Appendix Figures 7–9).

We document the emergence of DENV-3 lineage III_B.3.2 in Luanda, Angola, where the lineage probably was introduced from the Americas in late 2022, followed by local transmission across Luanda Province. Seasonal detection patterns aligned with climatic suitability for *Aedes* mosquito–borne transmission.

The emergence of DENV-3 in Angola raises concerns about disease severity given prior circulation of DENV-1 and DENV-2. In the absence of large-scale vaccination or vector-control programs, strengthening laboratory and clinical surveillance will be critical for outbreak detection and patient management (*6*). The risk extends beyond Luanda Province, which accounts for 27% of the country’s 38 million residents (https://data.worldbank.org/indicator/SP.POP.TOTL?locations=AO). *Ae. aegypti* mosquitos are widespread in the country (*7*), and climate projections indicate increasingly intense wet seasons in coastal Angola (*8*), further increasing the risk for arboviral transmission.

Given Angola’s history of *Aedes* mosquito–borne outbreaks, including yellow fever (2015–2016) (*9*) and Zika virus (2016–2017) (*10*), investment in laboratory capacity, capacity retention, and vector surveillance is urgent. Improved preparedness will help to mitigate the risk for sustained DENV transmission and related public health consequences.

Appendix Additional information about emergence of dengue virus serotype 3, lineage III_B.3.2, Angola.
