# Supplementary material for: Emergence of Dengue Virus Serotype 3, Lineage III_B.3.2, Angola
Source: Emerg Infect Dis. 2025 Nov;31(11):2194–7. doi: 10.3201/eid3111.251079 (PMC12704525; doi:10.3201/eid3111.251079)
Supplement: Appendix — Additional information about emergence of dengue virus serotype 3, lineage III_B.3.2, Angola. [file 25-1079-Techapp-s1.pdf]

Article DOI: https://doi.org/10.3201/eid3111.251079

EID cannot ensure accessibility for supplementary materials supplied by authors. Readers who have difficulty accessing supplementary content should contact the authors for assistance.

Emergence of Dengue Virus Serotype 3, Lineage III_B.3.2, Angola

Appendix

Materials and Methods

Real-time RT-qPCR for dengue, chikungunya, and Zika viruses

Serum samples were collected from 136 randomly selected from a convenience sample of individuals testing negative for malaria and presenting with suspected arbovirus infection that were tested locally for routine diagnostic using the SD. Bioline Dengue Duo NS1 Ag/IgG/IgM RDT assay, following the manufacturer's instructions. Viral RNA was extracted from all patient serum samples using the QIAamp Viral RNA Mini Kit (Qiagen, Germany), following the manufacturer's instructions. The extracted RNA was then subjected to reverse transcription quantitative PCR (RT-qPCR) using the TaqMan Arbovirus Triplex Kit (Thermo Fisher Scientific, UK), which allows simultaneous detection of dengue virus (DENV), chikungunya virus (CHIKV), and Zika virus (ZIKV) (*1*) and the TaqMan Arbovirus Triplex Control Kit as a positive control. Amplification was carried out on an ABI 7500 Fast Real-Time PCR system (Applied Biosystems, USA), and the results were interpreted according to the cycle threshold (Ct) criteria for each viral target. Samples were considered positive if Ct values were ≤38, indeterminate between >38 and ≤40, and negative if no amplification occurred or Ct >40. A summary of the number of tested cases per month, along with RT-qPCR results are shown in Appendix Figure 1.

Genome sequencing using the DENV pan-serotype primer set and analysis

Genome sequencing was performed on 16 DENV-positive samples identified by real-time RT-qPCR, using a multiplex-PCR amplicon-based approach, adapted from a previously described protocol (*2*). Specifically, a 350-bp "pan-serotype" DENV primer set was designed using thermodynamic modeling to amplify all four dengue virus serotypes. New primer set is

available at: https://labs.primalscheme.com/detail/artic-pan-dengue/400/v1.0.0/?q =). MinION sequencing libraries were prepared using the EXP-NBD114.96 Native Barcoding Kit (Oxford Nanopore Technologies [ONT], UK) and sequenced on a MinION device using R10.4.1 flow cells (ONT, UK). Barcoded read mapping and lineage identification were performed in real time using Rampart v1.2.0 (*3*), using DENV1–4 reference genomes (GenBank accession numbers: NC_001477, NC_001474, NC_001475, and NC_002640, respectively). Following identification of DENV3, consensus genome sequence reconstruction was performed with the ViralUnity workflow (*4*). Briefly, barcoded FASTQ files were aligned to the DENV3 reference genome using Minimap2 v2.22-r1101 (*5*). The resulting mapping files were converted to the BAM format, sorted and indexed with SAMtools v1.17 (*6*), which was also used to infer consensus genome sequences. Genome regions with coverage below 10× were masked. Genome coverage and assembly metrics were calculated using Bedtools v2.30.0 (*7*). Detailed assembly statistics are provided in Appendix Table. Correlation between RT-qPCR cycle thresholds (CT) and genome coverage is shown in Appendix Figure 2.

Collation of nucleotide datasets for primer design

Reference DENV genome sequences were downloaded from the GISAID EpiArbo database (*8*,*9*), as available on November 22, 2024. Only sequences with complete collection dates from November 2014 onward were considered. These were chosen to represent the past ten years of dengue virus diversity. Further, only complete coding sequences longer than 10,000 bp and with less than 25% ambiguous characters were retained. For each serotype, sequences were aligned using MAFFT v7.526 (*10*) and manually trimmed to remove untranslated regions. Maximum-likelihood phylogenetic trees were inferred using IQ-TREE v2.4.0 (*11*). Temporal signal assessment was then performed using TempEst v1.5.3 (*12*), and outliers in serotype-specific root-to-tip regressions were excluded. Final datasets for each serotype were registered with episets on GISAID, using the following identifiers: EPI_SET_250513 gp (DENV1, n = 6,482), EPI_SET_250513na (DENV2, n = 4,252), EPI_SET_250513ke (DENV3, n = 1,052), EPI_SET_250513ez (DENV4, n = 334).

Collation of nucleotide datasets for phylogenetic analyses

Complete and near-complete DENV3 genomes (minimum 7,500 bp, ≈70% of the genome of DENV3 reference genome (GenBank accession number: NC_001475) were downloaded from NCBI Viral Genome Resource (*13*), alongside available metadata associated with each sequence,

including date and location of sample collection and travel history. A total of 1,650 DENV3 sequences (sampling date range: 1953 to 2024), including the 6 new sequences generated in this study, were typed according to the most updated DENV lineage nomenclature (*14*) both with NextClade (*15*) and a recent alignment-free viral sequence classification tool (*16*). Overall, we observed a 94.12% (n = 1553/1650) agreement between the two classification tools at the minor-lineage level, and 83.33% (n = 5/6) agreement in the minor lineage classification of the Angolan sequences (classified as either DENV3 III_B3.2 or DENV3 III_B3), likely due to the small genome size of Angolan sequences. No disagreement between the two classification systems was observed at the genotype and major lineage levels.

Maximum likelihood phylogenetic analyses

DENV3 genotype III sequences from major lineage B (n = 1,100) were selected for phylogenetic analyses. Sequences were aligned using MAFFT v7.526 (*10*) and manually trimmed to remove untranslated regions. Five sequences from Angola (coverage >20%) were used for phylogenetic analyses. Preliminary maximum-likelihood phylogenetic trees were inferred using IQ-TREE v2.4.0 (*11*). Temporal signal assessment was then performed using TempEst v1.5.3 (*12*), and outliers in root-to-tip regressions were excluded, as described above. The final dataset comprised 1,013 DENV3_III sequences. Final maximum likelihood phylogenetic inference reconstruction was conducted using IQ-TREE v2.4.0 (*11*) with a best-fit model best nucleotide substitution model (*17*), and 1000 ultrafast bootstrap replicates to measure branch support (*18*) (-m MFP -bb 1000). A strong correlation between sampling time and genetic divergence (R^2 = 0.83) shows a strong temporal signal in our dataset (Appendix Figure 3), as expected for a relatively large dataset (n = 1,013) of dengue virus complete and near-complete genomes collected over 19.4 years. It also shows that Angola sequences, despite being near complete and partial, are not incongruent with their genetic diversity and phylogenetic position. We next used a phylogenetic molecular clock approach to explore the date of emergence of DENV3 in Angola. We inferred DENV evolutionary history in BEAST X (*19*) with BEAGLE v3 (*20*) under a HKY nucleotide substitution model (*21*) with a gamma distribution for rate heterogeneity, an uncorrelated molecular clock model (*22*), drawing branch rates from a lognormal distribution and a flexible non-parametric skygrid model (*23*) (with 59 grid points; cutoff 30 years). An ML tree was used as starting tree. MCMC was run for 100 million steps,

sampling trees and evolutionary parameters every 10,000 steps. Maximum clade credibility trees are shown in Appendix Figures 4,5.

Aedes aegypti **suitability**

Global hourly data for 2m dew point temperature and 2m mean temperature between 1 January 2023 to 31 December 2025 were downloaded from the ERA5-Land reanalysis dataset (ERA5-Land hourly data from 1950 to present) provided by Copernicus Climate Change Service at 0.1 × 0.1 degrees resolution (*24*). Relative humidity (RH) was calculated using the dew point (d) and temperature (T) following the August-Roche-Magnus formula

$$RH = 100 * \frac{\exp\,(d * 17.625)/(d + 243.04)}{\exp\,(T * 17.625)/(T + 243.04)}.$$

The points within Luanda province were sampled, categorised using administrative level 1 borders specified by a GADM shapefile (*25*). The surface area of each point grid square, which varies by latitude, was used to calculate the weighted average of the mean daily temperature and mean daily relative humidity.

IndexP, a measure of *Aedes* transmission potential, was calculated using the temperature and humidity data and the MVSE (Mosquito-borne Viral Suitability Estimator) R package (*26*,*27*). MVSE estimates indexP using a mechanistic transmission model implemented in a Bayesian Markov Chain Monte Carlo (MCMC) framework. The following priors were assumed for the model parameters, mosquito life expectancy (days): mean 14 and standard deviation 3, mosquito incubation period (days): mean 7 and standard deviation 2, mosquito biting frequency (bites/female/day): mean 0.25 and standard deviation 0.05, human life expectancy (years): mean 73 and standard deviation 2, human incubation period (days): mean 5 and standard deviation 1, human infectious period (days): mean 5 and standard deviation 1, and human-mosquito transmission probability: mean 0.5, standard deviation 0.01. All parameters were assumed to follow a Gaussian distribution. MCMC was run for 100000 steps and the posterior distributions were sampled 1000 times. Daily index P estimates were aggregated to calculate monthly averages for each year. The 2023–2025 estimates were further averaged to give the January to December seasonal curve in an average year and are shown in the context of confirmed cases in Appendix Figure 1.

Air travel data

Flight volumes to Angola were obtained from the International Air Transport Association (IATA). Calculated passenger volumes at the individual airport level were aggregated at the country level, focusing on countries where III_B3.2 lineage has been detected. For these, we compiled the mean number of passengers arriving in Angola between 2012 and 2021 (Appendix Figure 6).

References

1. Panmei K, Syed AH, Okafor O, Mammen S, Abraham AM. Performance evaluation of TaqMan™ Arbovirus Triplex Kit (ZIKV/DENV/CHIKV) for detection and differentiation of dengue and chikungunya viral RNA in serum samples of symptomatic patients. J Virol Methods. 2025;332:115072. PubMed https://doi.org/10.1016/j.jviromet.2024.115072

2. Quick J, Grubaugh ND, Pullan ST, Claro IM, Smith AD, Gangavarapu K, et al. Multiplex PCR method for MinION and Illumina sequencing of Zika and other virus genomes directly from clinical samples. Nat Protoc. 2017;12:1261–76. PubMed https://doi.org/10.1038/nprot.2017.066

3. Hadfield J. artic-network/rampart: read assignment, mapping, and phylogenetic analysis in real time, version 1.2.0 [cited 2025 Feb 1]. https://github.com/artic-network/rampart

4. Romero FR. ViralUnity. 2025 [cited 2025 Feb 1]. https://github.com/filiperomero2/ViralUnity

5. Li H. Minimap2: pairwise alignment for nucleotide sequences. Bioinformatics. 2018;34:3094–100. **PMID 29750242**

6. Li H, Handsaker B, Wysoker A, Fennell T, Ruan J, Homer N, et al.; 1000 Genome Project Data Processing Subgroup. The Sequence Alignment/Map format and SAMtools. Bioinformatics. 2009;25:2078–9. PubMed https://doi.org/10.1093/bioinformatics/btp352

7. Quinlan AR, Hall IM. BEDTools: a flexible suite of utilities for comparing genomic features. Bioinformatics. 2010;26:841–2. PubMed https://doi.org/10.1093/bioinformatics/btq033

8. Wallau GL, Abanda NN, Abbud A, Abdella S, Abera A, Ahuka-Mundeke S, et al.; Global Arbovirus Researchers United. Arbovirus researchers unite: expanding genomic surveillance for an urgent global need. Lancet Glob Health. 2023;11:e1501–2. PubMed https://doi.org/10.1016/S2214-109X(23)00325-X

9. Shu Y, McCauley J. GISAID: Global initiative on sharing all influenza data - from vision to reality. Euro Surveill. 2017;22:30494. PubMed https://doi.org/10.2807/1560-7917.ES.2017.22.13.30494

10. Katoh K, Misawa K, Kuma K, Miyata T. MAFFT: a novel method for rapid multiple sequence alignment based on fast Fourier transform. Nucleic Acids Res. 2002;30:3059–66. PubMed https://doi.org/10.1093/nar/gkf436

11. Minh BQ, Schmidt HA, Chernomor O, Schrempf D, Woodhams MD, von Haeseler A, et al. IQ-TREE 2: new models and efficient methods for phylogenetic inference in the genomic era. Mol Biol Evol. 2020;37:1530–4. PubMed https://doi.org/10.1093/molbev/msaa015

12. Rambaut A, Lam TT, Max Carvalho L, Pybus OG. Exploring the temporal structure of heterochronous sequences using TempEst (formerly Path-O-Gen). Virus Evol. 2016;2:vew007. PubMed https://doi.org/10.1093/ve/vew007

13. Brister JR, Ako-Adjei D, Bao Y, Blinkova O. NCBI viral genomes resource. Nucleic Acids Res. 2015;43(D1):D571–7. PubMed https://doi.org/10.1093/nar/gku1207

14. Hill V, Cleemput S, Pereira JS, Gifford RJ, Fonseca V, Tegally H, et al. A new lineage nomenclature to aid genomic surveillance of dengue virus. PLoS Biol. 2024;22:e3002834. PubMed https://doi.org/10.1371/journal.pbio.3002834

15. Aksamentov I, Roemer C, Hodcroft E, Neher R. Nextclade: clade assignment, mutation calling and quality control for viral genomes. J Open Source Softw. 2021;6:3773. https://doi.org/10.21105/joss.03773

16. van Zyl DJ, Dunaiski M, Tegally H, Baxter C, de Oliveira T, Xavier JS, et al.; INFORM Africa research study group. Alignment-free viral sequence classification at scale. BMC Genomics. 2025;26:389. PubMed https://doi.org/10.1186/s12864-025-11554-5

17. Kalyaanamoorthy S, Minh BQ, Wong TKF, von Haeseler A, Jermiin LS. ModelFinder: fast model selection for accurate phylogenetic estimates. Nat Methods. 2017;14:587–9. PubMed https://doi.org/10.1038/nmeth.4285

18. Minh BQ, Nguyen MAT, von Haeseler A. Ultrafast approximation for phylogenetic bootstrap. Mol Biol Evol. 2013;30:1188–95. PubMed https://doi.org/10.1093/molbev/mst024

19. Suchard MA, Lemey P, Baele G, Ayres DL, Drummond AJ, Rambaut A. Bayesian phylogenetic and phylodynamic data integration using BEAST 1.10. Virus Evol. 2018;4:vey016. PubMed https://doi.org/10.1093/ve/vey016

20. Ayres DL, Cummings MP, Baele G, Darling AE, Lewis PO, Swofford DL, et al. BEAGLE 3: improved performance, scaling, and usability for a high-performance computing library for

statistical phylogenetics. Syst Biol. 2019;68:1052–61. PubMed https://doi.org/10.1093/sysbio/syz020

21. Hasegawa M, Kishino H, Yano T. Dating of the human-ape splitting by a molecular clock of mitochondrial DNA. J Mol Evol. 1985;22:160–74. PubMed https://doi.org/10.1007/BF02101694

22. Drummond AJ, Ho SYW, Phillips MJ, Rambaut A. Relaxed phylogenetics and dating with confidence. PLoS Biol. 2006;4:e88. PubMed https://doi.org/10.1371/journal.pbio.0040088

23. Gill MS, Lemey P, Faria NR, Rambaut A, Shapiro B, Suchard MA. Improving Bayesian population dynamics inference: a coalescent-based model for multiple loci. Mol Biol Evol. 2013;30:713–24. PubMed https://doi.org/10.1093/molbev/mss265

24. Carslaw D. worldmet: import surface meteorological data from NOAA Integrated Surface Database (ISD). 2017 [cited 2025 May 28]. https://CRAN.R-project.org/package=worldmet

25. Muñoz Sabater J. ERA5-Land hourly data from 1950 to present. Copernicus Climate Change Service climate data store. 2019 [cited 2025 Feb 1]. https://cds.climate.copernicus.eu/datasets/reanalysis-era5-land?tab=overview

26. GADM. Global Administrative Areas. GADM database of Global Administrative Areas, version 2.0. 2018 [cited 2025 Feb 1]. https://www.gadm.org

27. Obolski U, Perez PN, Villabona-Arenas CJ, Thézé J, Faria NR, Lourenço J. MVSE: an R-package that estimates a climate-driven mosquito-borne viral suitability index. Methods Ecol Evol. 2019;10:1357–70. PubMed https://doi.org/10.1111/2041-210X.13205

28. Rambaut A. FigTree v1.4.4 [cited 2025 Feb 1]. http://tree.bio.ed.ac.uk/software/figtree

Appendix Table. Clinical, demographic, and sequencing data for NS1-positive suspected dengue virus infections, Luanda Province, Angola, 2024*

ID	Date	dT	Location	Sex	Age	NS1	Ct	# mapped reads	Depth	Coverage
1	02/06/2024	0	Zango	M	67	+	19.6	7771	277	91.9
2	07/04/2024	0	Luanda	M	38	+	27.1	1902	60	59.6
3	01/06/2024	1	Camama	F	13	+	25.9	1071	36	49.6
4	24/07/2024	4	Viana	F	9	+	31.6	579	23	25.6
5	12/04/2024	5	Luanda	F	39	+	32.3	169	6	20.1
6	31/07/2024	0	Luanda	F	48	Neg	28.8	896	36	15.8
7	12/04/2024	2	Luanda	F	74	+	30.6	143	5	11.6
8	04/06/2024	0	Luanda	M	35	+	26.8	103	4	4.4
9	11/05/2024	3	Luanda	M	28	+	28.8	79	3	4.2
10	05/06/2024	1	Viana	M	8	+	25.2	97	3	2.0
11	12/07/2024	3	Luanda	M	39	+	32.5	64	2	0.9
12	09/06/2024	1	Luanda	F	9	NA	26.9	33	1	0.0
13	03/06/2024	4	Zango	F	11	+	31.5	30	1	0.0
14	28/05/2024	3	Luanda	F	0	+	32.1	18	0.73	0.0
15	30/05/2024	0	Zango	F	24	–	36.8	17	0.67	0.0
16	28/05/2024	†	Viana	–	45	NA	32.6	NA	NA	0.0

*Ct-values are from RT-qPCR. Sequencing metrics include number of DENV mapped reads, average depth, and genome coverage (%) against DENV3 reference strain. NA, not tested for DENV. Samples with IDs 1–5 were included in phylogenetic analyses. RT-PCR positive (including sequenced) samples were obtained from three of the nine municipalities in the Province of Luanda. Zango is a large urban district within the municipality of Viana, and ≈32 Km from Luanda city center. Camama is a neighborhood within Talatona municipality, and ≈111 Km from Luanda city center. Viana is a municipality within Luanda Province and around 18 Km from Luanda city center.

†Sample was collected within the scope of the Occupational Medicine Service (patient with no symptoms). Sample IDs 2 and 11 pertain to patients admitted to hospital with thrombocytopenia and required platelet transfusion. dT corresponds to the number of days between symptom onset and sample collection.

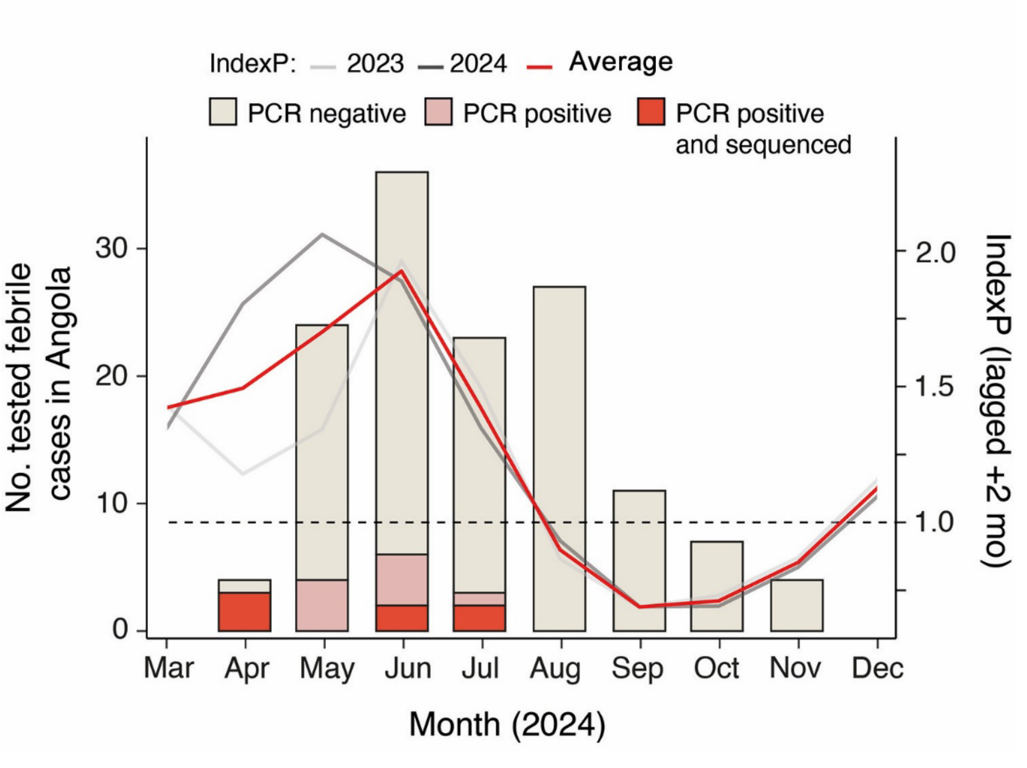

Appendix Figure 1. Monthly distribution of tested febrile cases in Luanda Province, Angola, 2024. Bars show RT-qPCR and sequencing results: PCR-negative (gray), PCR-positive (light red), and PCR-positive with sequencing (dark red). Lines indicate the IndexP climatic suitability index for *Aedes aegypti* transmission (lagged by 2 months), with gray representing 2023, black 2024, and red the multi-year average.

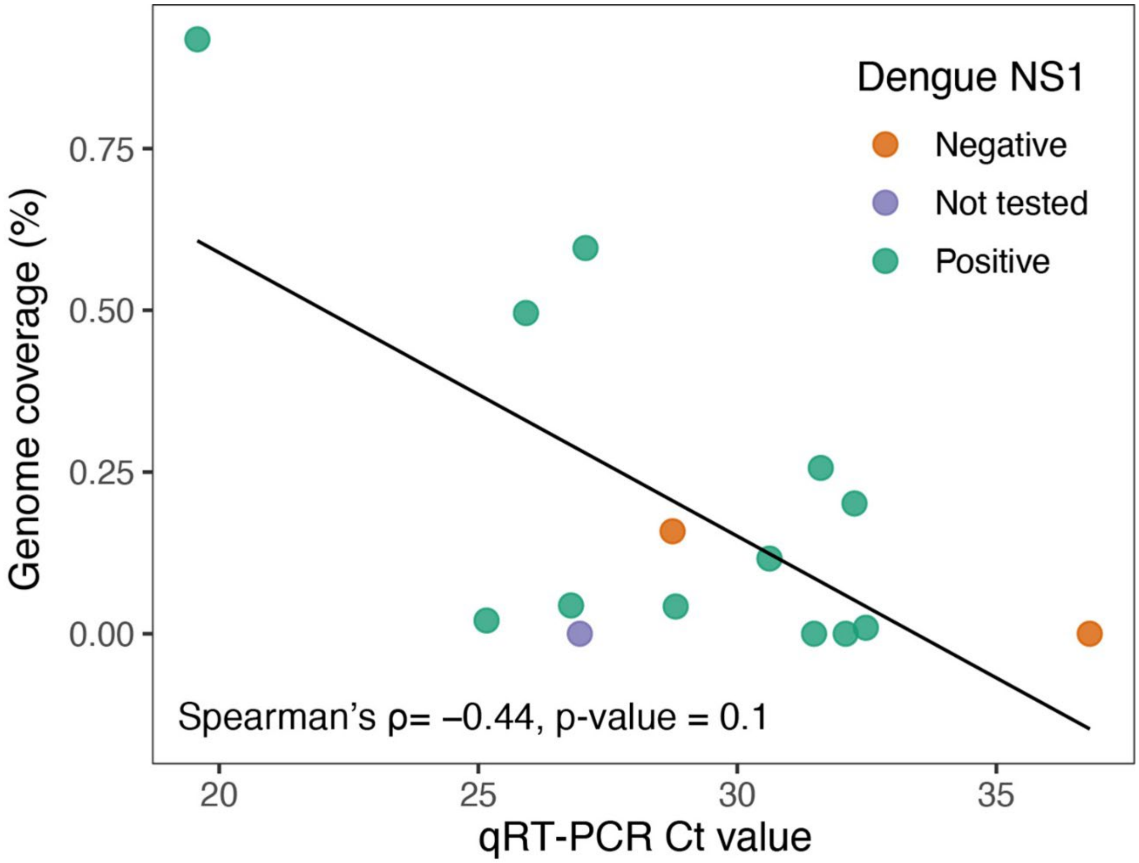

Appendix Figure 2. Correlation between RT-PCR cycle thresholds (Ct) and DENV horizontal genome coverage. Data points are color-coded based on NS1 status: positive (green), negative (orange), and not available (gray). Line indicates a significant negative relationship, with a Spearman correlation coefficient of –0.44 and a p-value of 0.1.

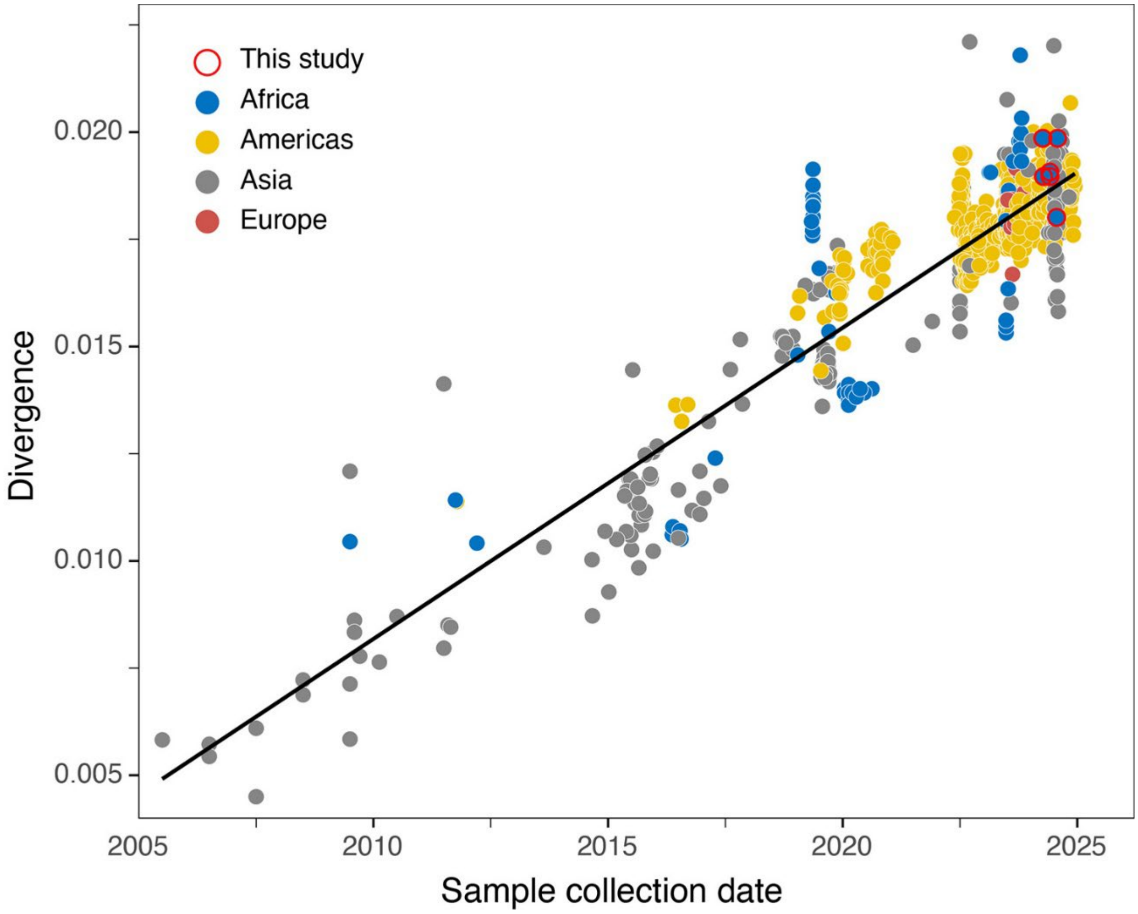

Appendix Figure 3. Root-to-tip regression of DENV 3III_B sequences (n = 1,013) over time. Data points show the relationship between genetic distance and sample collection dates for DENV 3III_B sequences from infections across four global regions. Each point represents a sequence, colored by its geographic origin: Africa (blue), Americas (yellow), Asia (gray) and Europe (red). Five sequences generated in this study (coverage >20%) are shown as blue circles highlighted with a red stroke.

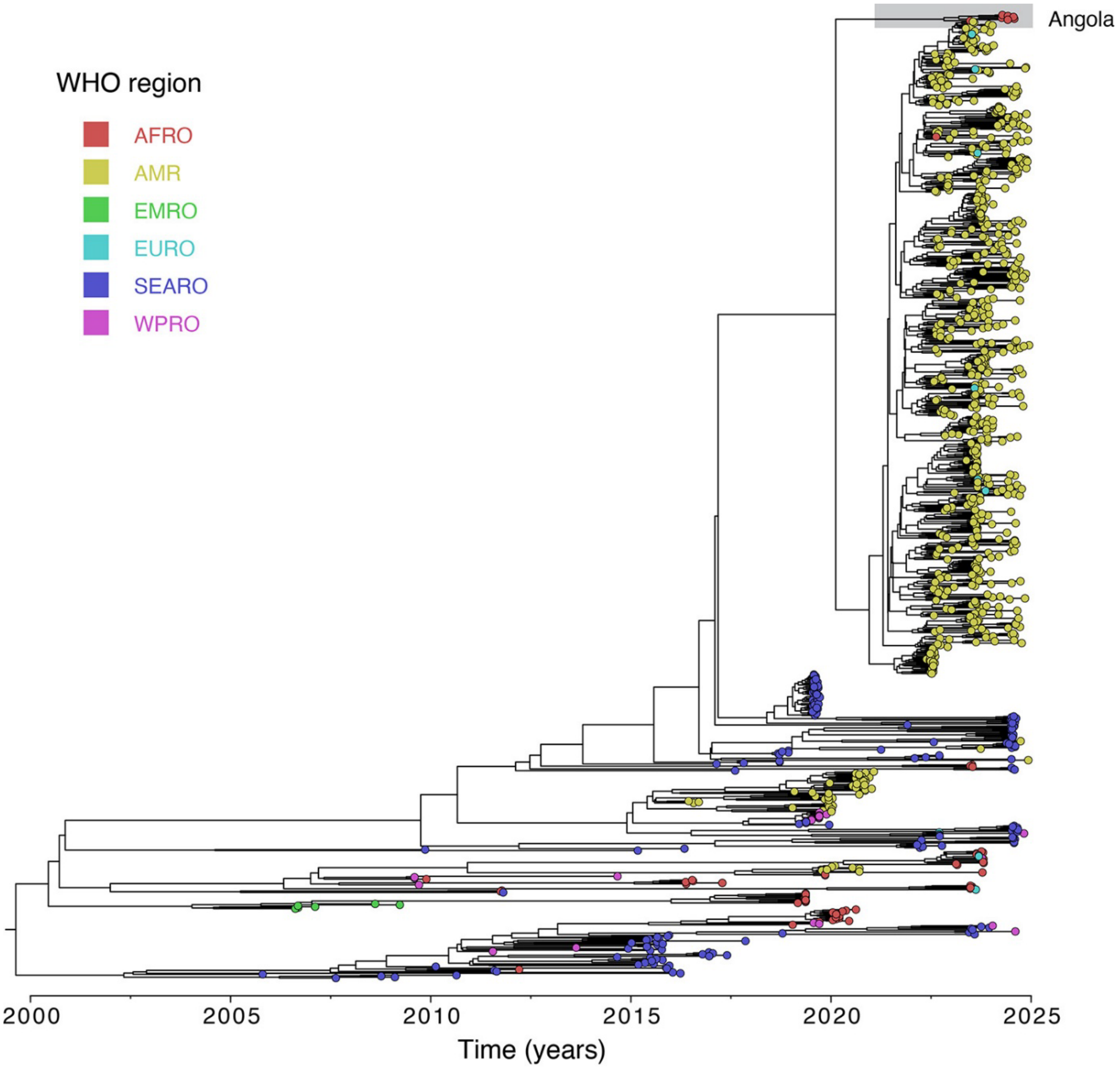

Appendix Figure 4. Maximum clade credibility tree generated from 1,013 DENV 3III_B lineage genomes. Tips are colored according to sampling region. Angolan clade (n = 5) is highlighted in a gray box.

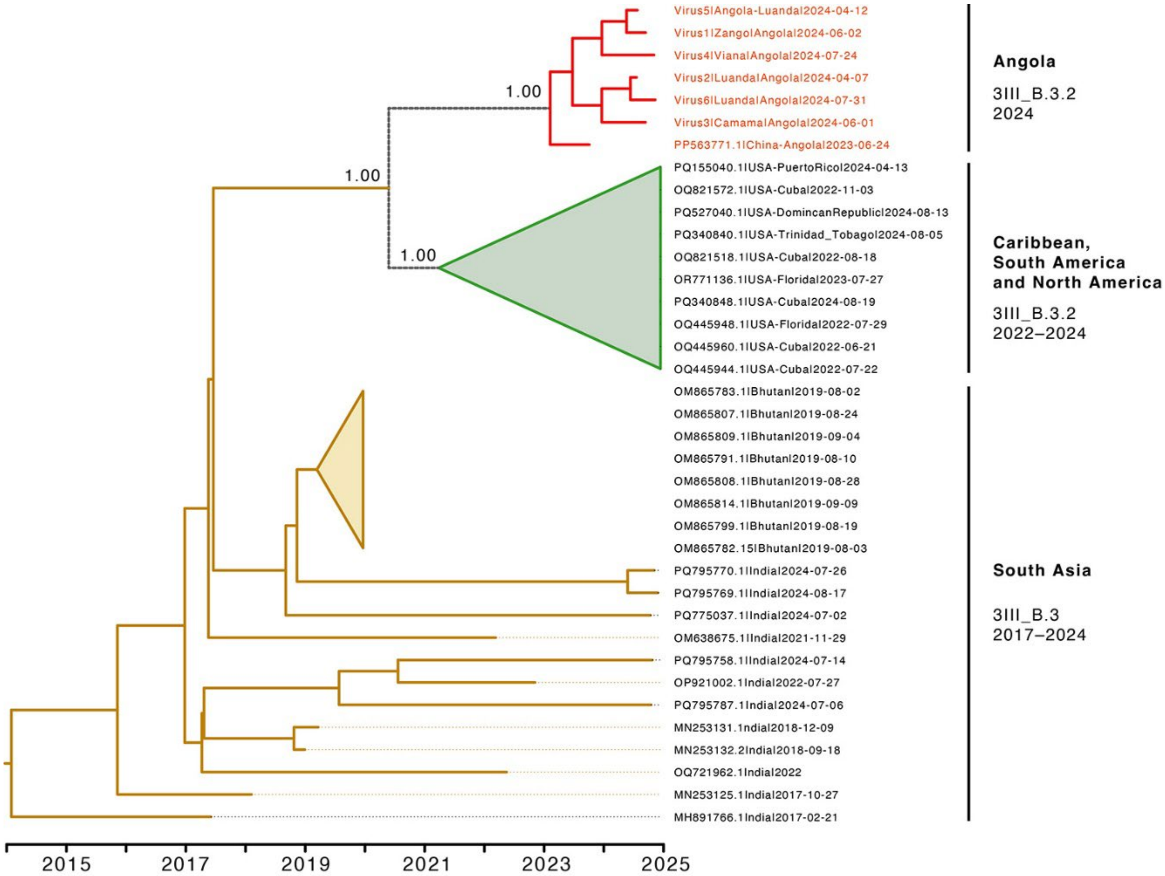

Appendix Figure 5. Subset of the maximum clade credibility tree shown in Appendix Figure 4. Branches are colored according to sampling region inferred using a standard parsimony approach implemented in FigTree v1.4.4 (*28*).

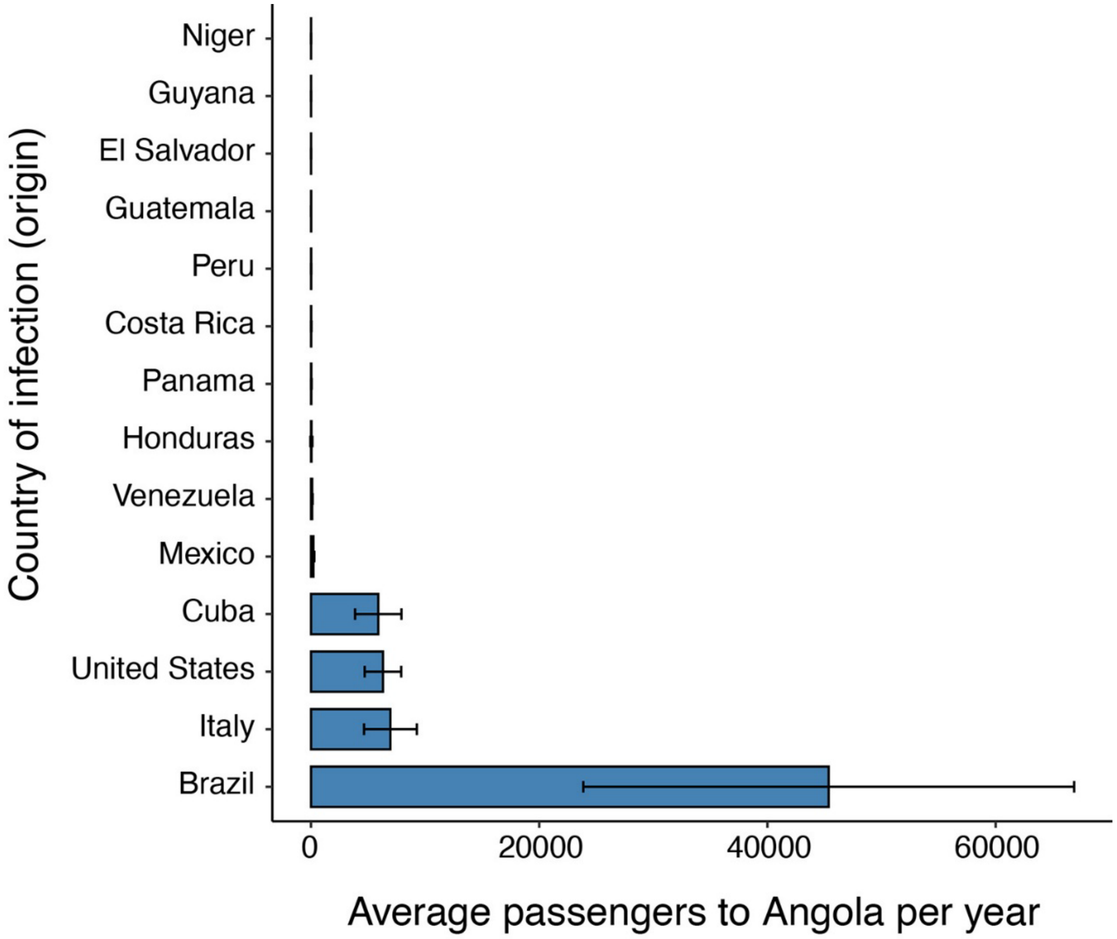

Appendix Figure 6. Average annual number of air passengers arriving in Angola between 2012–2021 from countries where DENV3III_B.3.2 lineage has been detected (see also Appendix Figure 8).

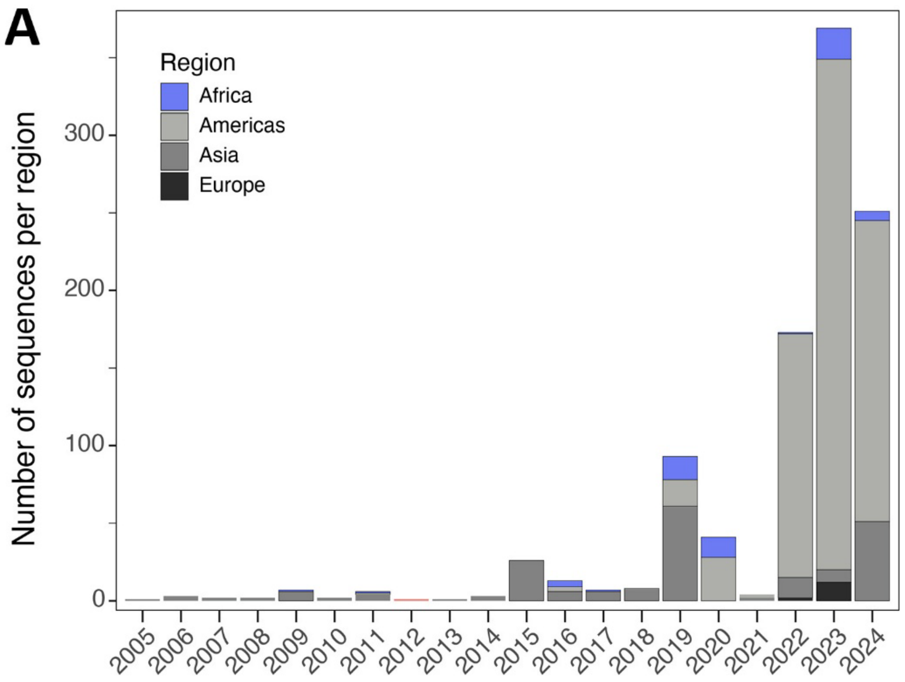

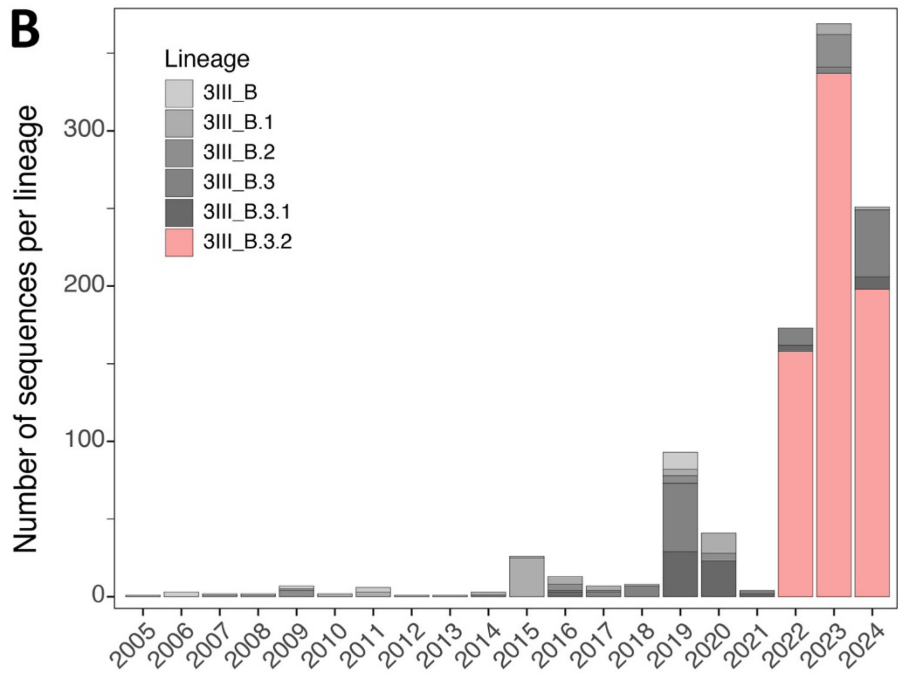

Appendix Figure 7. Number of DENV3 III_B sequences globally (n = 1,013 sequences) per year stratified by geographic region (a) and lineage (b). Upper panel highlights limited availability of DENV3_B sequences from Africa (n = 63/1,013), while lower panel highlights the recent boom of 3III_B.3.2 minor lineage. We identified only eight 3III_B.3.2 sequences from Africa: seven from Angola (six identified in this study), and one from Niger identified in 2022 (Accession Number OQ132878.1).

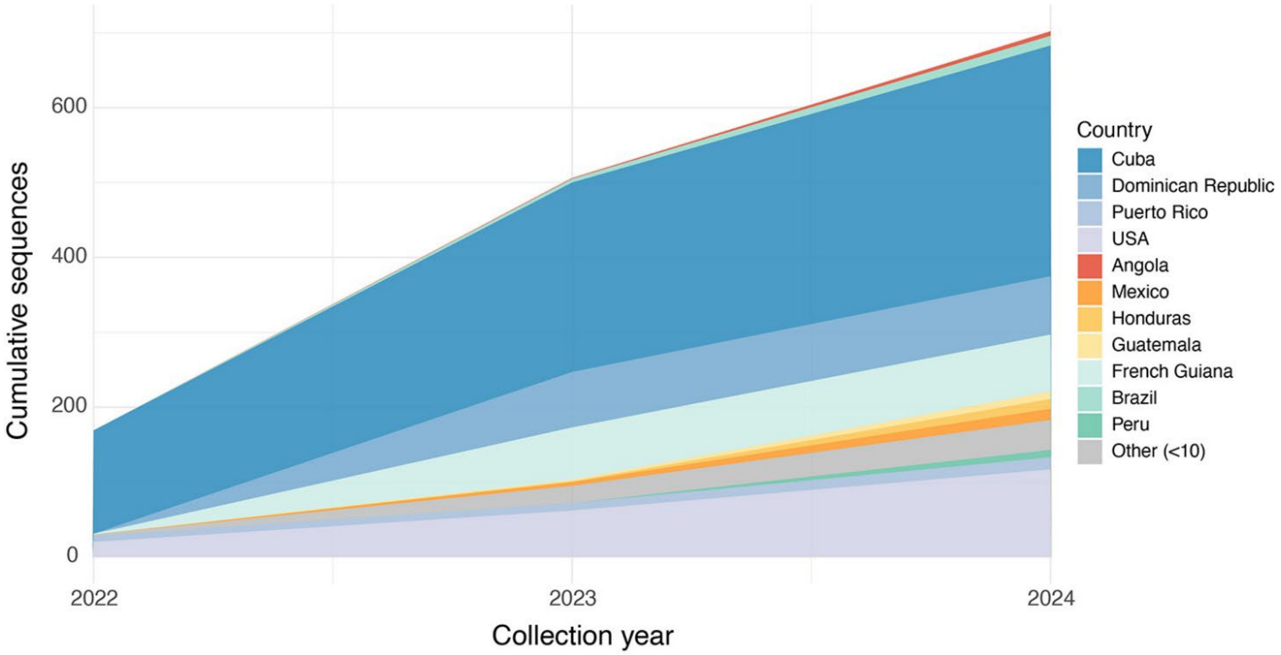

Appendix Figure 8. Cumulative number of DENV3III_B3.2 lineage sequences by country and year of sample collection. Countries with <10 sequences are grouped as “Other” and include Costa Rica (n = 7), Trinidad and Tobago (n = 7), Haiti (n = 4), Guyana (n = 3), Italy (n = 2), El Salvador (n = 2), Saint Lucia (n = 1), Saint Martin (n = 1), Panama (n = 1), Niger (n = 1) and Venezuela (n = 1).

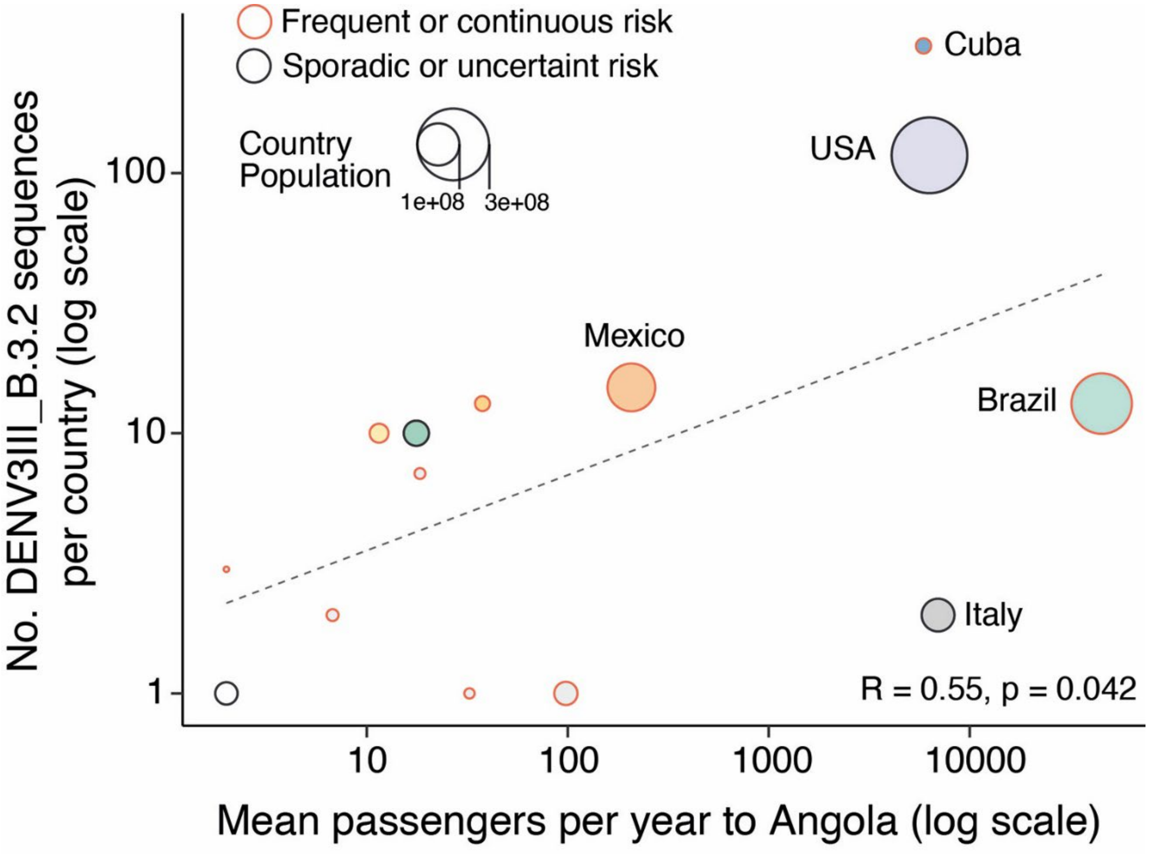

Appendix Figure 9. Correlation between DENV 3III_B.3.2 lineage sequence counts per country (log scale) and the mean annual passenger volume to Angola (log scale), highlighting countries with frequent or uncertain dengue transmission risk.
